# Animal bites presenting in the pediatric emergency department at Al Qassimi Women's and Children's Hospital: a cross-sectional study

**DOI:** 10.11604/pamj.2025.52.91.44242

**Published:** 2025-11-03

**Authors:** Batool Zaffar Ali, Nader Francis, Amreen Sajith, Amal Sherif, Safiya Saif, Khurshid Khan, Layla Taryam, Sinan Yavuz

**Affiliations:** 1 Department of Pediatrics, Al Qassimi Women's and Children's Hospital, Sharjah, United Arab Emirates

**Keywords:** Animal bites, pediatrics, emergency department, rabies post-exposure prophylaxis, rabies vaccine

## Abstract

Animal bites represent a major public health concern that frequently goes underreported. Dogs and cats are the most common culprits. This study aims to gather comprehensive data on patients with animal bites who visited Al Qassimi Women's and Children's Hospital (AQWCH) in the United Arab Emirates (UAE). The research method involves a retrospective chart review of children under 13 years old who visited the hospital's Emergency Department (ED) with a history of animal bites between January 2018 and September 2023. The study aims to identify the high-risk groups for such incidents and provide valuable insights into demographics, types, and locations of animal bites, as well as clinical presentation, management, and outcomes of patients. The majority of patients were male 708 (58%), and domestic animals 701 (72%), particularly cats 1003 (82.08%), were the primary cause of bites. Scratches were the most common type of injury, 942 (77.21%), and often occurred after provocation, 95.5%, with the right upper limb being the most affected, 492 (44.40%). The majority of patients received the rabies vaccine 1030 (84%), and no cases of rabies were recorded during the study period. In conclusion, cat and dog bites make up the majority of animal bites, and the study provides crucial insights into the characteristics and patterns of such incidents.

## Introduction

Animals play a crucial role in the ecosystem. Animal bites impose a significant burden on the global healthcare system. In the United States, animal bites account for one percent of annual emergency department (ED) visits. Injuries resulting from animal bites range from minor to fatal. It is essential to promptly assess all animal bites due to the potential for severe and fatal complications. The majority of animal bites occur in domestic settings, with dogs and cats being the most common culprits [1,2]. The most dangerous animal bites typically come from snakes, dogs, cats, and monkeys. Consequences of these injuries can include unsightly soft tissue and skeletal damage, scarring, and disfigurement. Children are more prone to animal bites compared to adults, and the most commonly bitten areas are the neck and head [3]. Rabies, a viral infection that can result from animal bites, has a high mortality and morbidity rate. It causes numerous deaths worldwide each year, with Asia and Africa being the most affected regions. Rabies is preventable through vaccination [1]. Immediate prehospital management involves washing the wound with soap or detergent and then flushing it with water for 15 minutes. After the water flush, iodine or antiviral medication should be applied. It is crucial to avoid using irritants or covering the wound. The biting animal should be observed for 10 days. Post-exposure prophylaxis (PEP) includes human rabies immune globulin (HRIG) and rabies vaccine, administered on days 0, 3, 7, and 14 [1]. The goal of this study is to describe the demographic features, types, and locations of animal bites, clinical presentations, management, and outcomes of patients diagnosed with animal bites at AQWCH. This study will be the first in the UAE to address such a topic specifically in the pediatric age group. Objective: The purpose of this study is to describe the demographic features, clinical presentation, types and locations of animal bites, management, and outcome of patients diagnosed with animal bites at AQWCH. The primary objectives were to collect and explore descriptive data of patients with animal bites who were presented to the tertiary hospital ED in the UAE. Secondary objectives were to explore children´s characteristics and factors associated with animal bites, common location of animal bites, the types of animal bites, to better understand if there is any seasonal occurrence, to recognize the presenting symptoms and signs of children with animal bites, and to improve knowledge of methods for managing children with an animal bite.

## Methods

**Study design:** this study is a cross-sectional study conducted at AQWCH in the United Arab Emirates (UAE). We analyzed de-identified medical records of all patients who presented to the emergency department with a history of animal bites between January 1^st^, 2018, and September 1^st^, 2023.

**Study setting:** this study was conducted at one site, AQWCH, in the UAE. Patients who visited AQWCH with a diagnosis of animal bites between January 1^st^, 2018, and September 1^st^, 2023, were included in their visit to AQWCH.

**Participants:** even though the research is based on a retrospective analysis, it was necessary to establish the study population through specific inclusion and exclusion criteria. Identification of cases was performed by examining diagnosis codes and selecting those within the correct age bracket. The study included all children under the age of 13 who were admitted during the specified period and had a final diagnosis of animal bites. Patients whose recorded data were absent from the electronic system were excluded from the study.

**Variables:** in this cross-sectional study, a data abstraction form was used for data collection from digital medical records, which are demographic (age, gender, duration of stay in the hospital), characteristics of animal bites (type of animal, type of injury, domestic animal or street animal, provoked, the site of injury, the date of bite prehospital visit), patient admission (inpatient, only ED visit), medical management (vaccinated against rabies and tetanus, number of rabies vaccine given, received prophylactic antibiotic, needed immunoglobulin), associated complications (fever, wound infection, systemic infection, bone fracture).

**The data source of the study:** the data source for this study was digital medical records of children who visited AQWCH with a diagnosis of animal bites during the specified time frame. We collected demographic information, dates of visit to the ED, clinical notes, discharge diagnoses, and surgical details from operative notes. Patients were identified using diagnosis codes and age ranges. All selected patients were minors, and their data were de-identified, anonymous, and stored on password-protected computers accessible only to the principal investigator. The cases were identified by checking the diagnosis code and the appropriate age range. The chart abstractor validated the inclusion/exclusion criteria in the data abstraction sheet before continuing with the abstraction. The study procedure was limited to a review of existing medical records.

**Measures to minimize bias:** the data abstractors were blind to the development of the study proposal and protocol. In other words, they were blind to the study objectives and research questions. This was done to reduce the observer and data abstractor bias in the study.

**Study size:** all patients who presented to the ED with a history of animal bite at AQWCH between January 1^st^, 2018, and September 1^st^, 2023, and fulfilled the inclusion and exclusion criteria were included in our study.

**Quantitative variables:** quantitative variables (age, number of rabies vaccine doses given, and the time of hospital visit) were described using means and standard deviation.

**Statistical analysis:** cases of animal bites that were presented at AQWCH from January 1^st^, 2018, to September 1^st^, 2023, were identified retrospectively using an electronic database from the Pediatric Department. All patients were identified using a computerized search of hospital discharge diagnoses, coded as “animal bites, scratches, bites due to dog, cat, camel, etc.” Children with missing recorded data were excluded. The baseline descriptive data were presented visually as tables, and the analyzed results were displayed visually as charts. Data from medical records were extracted into a single Excel spreadsheet database, which was checked for errors and missing data. Descriptive statistics of means for continuous data, frequency and percentage for categorical data were used to analyze demographics (gender, duration of stay in the hospital), characteristics of animal bites (type of animal, type of injury, domestic animal or street animal, provoked, the site of injury), patient admission (inpatient, only ED visit), medical management (vaccinated against rabies and tetanus, received prophylactic antibiotics, needed immunoglobulin), and associated complications (fever, wound infection, systemic infection, bone fracture). We used SPSS for analysis in our study.

**Ethical consideration:** this study is a cross-sectional study. All selected patients are minors; however, all data were unidentified, anonymous, and stored on password-protected computers accessed by the Primary Investigator (PI) only. Ethical approval for this study was obtained from MOHAP REC (Approval Number: MOHAP/DXB-REC/O.O. O/No.133/ 2023). The PI has requested a waiver of informed consent (parental permission) and a waiver of assent (minor participants) as this study met the following criteria: i) the research was limited to the existing data and involved no more than minimal risk to the participants; ii) the waiver was adversely affecting the rights and welfare of the participants; ii) the study could not be feasible without the waiver.

## Results

**Figure 1:**
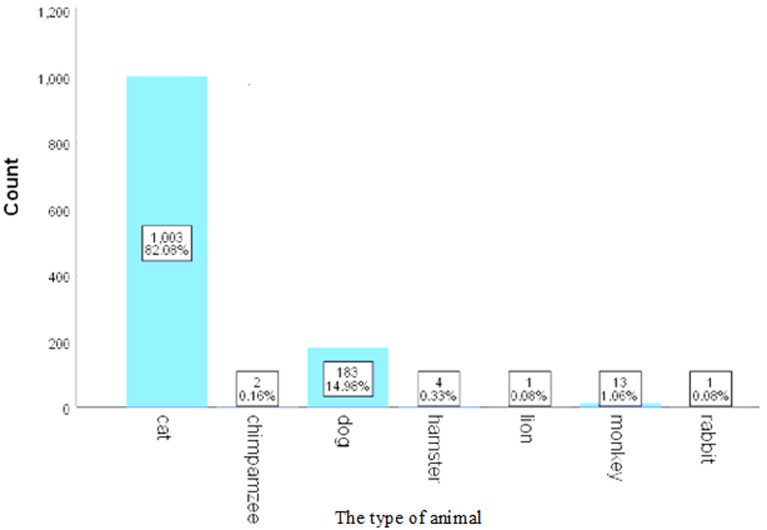
type of animal

**Figure 2:**
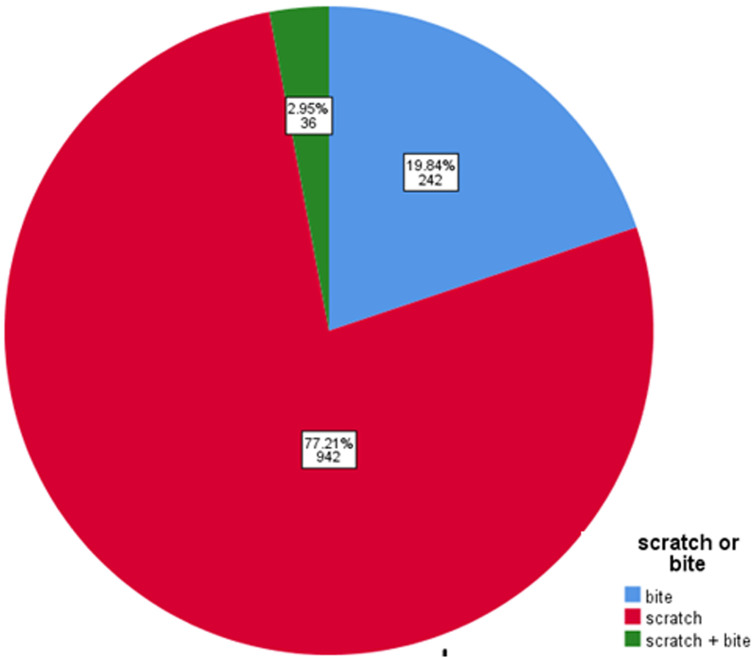
type of injury

The total number of patients who visited the emergency department was 1225. Out of these, 708 (58%) were male and 518 (42%) were female, with a mean age of 5.7 years and a standard deviation of 3.3 years. Of the injuries, 701 (72%) were caused by domestic animals and 524 (38%) by street animals, with 463 (95.5%) of the incidents being provoked. The average duration before visiting the hospital after being bitten was 1.3 days, with an SD of 1 day. Rabies vaccine was administered to 1030 (84%) of the patients, with an average of 3.5 doses given and an SD of 0.9. Additionally, 35 (3%) patients received the tetanus vaccine, 238 (19.5%) were given antibiotics, and 15 (1.2%) received immunoglobulin (Table 1). The most frequently encountered animal was a cat, with 1003 instances (82.08%). This was followed by dogs at 153 (14.98%), rats at 14 (1.15%), monkeys at 13 (1.06%), hamsters at 4 (0.33%), chimpanzees at 2 (0.16%), lions at 1 (0.08%), turtles at 1 (0.08%), and rabbits at 1 (0.08%) (Figure 1). The predominant type of injury was a scratch, occurring in 942 cases (77.21%), followed by bites in 242 cases (19.84%), and simultaneous scratches and bites in 36 cases (2.95%) (Figure 2). During the injury site testing, we observed that the most common injury occurred in the upper right limb, with 492 cases (44.40%), followed by the left upper limb with 237 cases (21.9%), the head with 127 cases (11.46%), the right lower limb with 112 cases (10.11%), the left lower limb with 65 cases (7.67%) and other with each of the following sites (abdomen, back, chest, neck, and combined injuries) (4.24%). In this study, none of the patients developed rabies, required hospital admission, or experienced bone fractures, systemic or local wound infections.

**Table 1:** characteristics of the children with an animal bite

Frequencies	Overall study
**Gender, no (%)**	
Male	708 (58)
Female	518 (42)
Age, years, mean (SD)	5.7 (3.3)
The time of hospital visit, days, mean (SD)	1.3 (1)
Provoked, no (%)	463 (95.5)
Domestic animal, no (%)	701 (72)
Rabies vaccine, no (%)	1030 (84)
Number of rabies vaccine doses given, days, mean (SD)	3.5 (0.96)
Tetanus vaccine given, no (%)	35 (3)
Antibiotic given, no (%)	238 (19.5)
Immunoglobulin given, no (%)	15 (1.2)

SD: standard deviation

## Discussion

In our study, we examined 1225 patients aged 2 to 5 years who visited the emergency department due to an animal bite. We aimed to gather and analyze descriptive data on patients with animal bites to understand the characteristics of the children affected, the factors associated with the bites, the common locations and types of animal bites, as well as the symptoms and signs presented by the children. Additionally, we sought to improve our understanding of the methods for managing children with animal bites and the outcomes in the UAE. Our study was cross-sectional. One limitation was the lack of references and articles on this topic in the UAE. Moreover, the study was conducted at a single center in the city center, which may not fully represent the entire country. Animals are an essential part of the human ecosystem. Animals may exhibit different behaviors toward humans, including loyalty, affection, or aggression. In the United Arab Emirates (UAE), information on this subject is limited, yet animal bites remain a significant public health concern. The only documented study in the UAE focused on camel bites specifically. Animal bites among children represent a serious health issue. Consistent with findings from other research, our study identified the average age of those affected as 5.7 years, with boys being more likely to sustain injuries [4-6]. We speculate that the higher incidence in males may be attributed to their behavioral tendencies, including more frequent outdoor activities and differences in the way they play. In the study presented, domestic animals accounted for 72% of bite cases, with 95.5% of these bites being provoked, aligning with previous literature [7]. Contrary to our findings, where cats comprised 82% of the total animals and scratches were the most common injury at 77.21% [8], we hypothesized that this reflects the prevalent types of adopted and street animals in the area. Unlike studies involving children [7], our research indicated that the upper right limb was the most frequently injured area.

The rabies virus is a lyssavirus species, and in the absence of vaccination, it is a mostly fatal disease. The virus can be transmitted by wild and domestic animals. When an infected animal bites, its saliva can transfer the disease through an open wound. Rabies is prevalent worldwide, with over 60,000 people dying from infection annually in Asia, resulting in over 1.2 million cases of physical disability. Africa and India follow closely in terms of the number of cases. Rabies is a notable disease in the Middle East, with an estimated 350 cases annually, most of which involve children. The majority of these cases are reported in Jordan, the Syrian Arab Republic, Lebanon, and the Islamic Republic of Iran. Post-exposure prophylaxis (PEP), including the rabies vaccine and human rabies immune globulin (HRIG), is administered following World Health Organization (WHO) guidelines. In our data, 84% of patients received a rabies vaccine, while 1.2% required HRIG. It remains unclear why some children did not receive PEP. In most of their electronic medical records, we found that parents had refused the treatment, but the reasons for this refusal were not documented. Animal bites can range from minor injuries to severe, life-threatening conditions or infections. Therefore, treatment should commence immediately at the scene by thoroughly washing the wound with soap and keeping it under running water for 15 minutes. When the patient arrives at the emergency department, careful management is necessary. Prophylactic antibiotics should be administered to prevent wound infection, and post-exposure prophylaxis (PEP) should be provided depending on the animal's status. Individuals travelling to areas where rabies is suspected should be provided with information about prophylaxis and encouraged to contact public health agencies for assistance [1,9]. Fortunately, in the current study, there were no cases of major trauma, bone fractures, systemic or local wound infections, or hospital admissions. Additionally, 19% of patients were prescribed antibiotics for prophylactic purposes. The UAE has a highly effective vaccination strategy for animals, significantly reducing animal-related diseases. For instance, all dogs must be vaccinated against rabies and have a microchip with registration. According to the Ministry of Health and Prevention's annual report in 2020, the incidence of rabies in the country was zero.

## Conclusion

In summary, our findings indicate that cats are the most frequent cause of animal bites, followed by dogs, with the majority of bites occurring in males. Most bites happen after provocation. Although no fatal injuries were observed, animal bites remain a significant public health issue. Post-exposure prophylaxis (PEP) is the cornerstone of treatment, alongside wound care. Emergency physicians should be knowledgeable about the type of animal and its bite to ensure prompt management. The UAE's strategies for prevention, public education, and the vaccination of all domestic animals, particularly dogs, have led to the successful eradication of rabies in the country.

### What is known about this topic

- Animal bites have a significant impact on global public health;
- The most common animal involved is dog followed by cat;
- The injury ranges from simple to life-threatening.

### What this study adds

- To our knowledge, this study was the first study in the United Arab Emirates that tackles animal bites in pediatric ages;
- This study revealed that the most common animal bites are from a cat, followed by a dog, in the United Arab Emirates;
- There were no fatal injuries reported, and no cases of rabies were reported either, indicating effective animal vaccination strategies and the easy accessibility of vaccines at local centers when needed.
